# Erratum

**Published:** 2013-01-11

## Vol. 61, Nos. 51 & 52

In the report, “Cervical Cancer Screening Among Women by Hysterectomy Status and Among Women Aged ≥65 Years — United States, 2000–2010,” an error occurred on page 1045, in the third sentence of the first paragraph of the Editorial Note. The sentence should read as follows: “Despite consistent guidelines by three national organizations (USPSTF, ACS, and ACOG) recommending against routine screening for cervical cancer posthysterectomy, the proportion of women aged **≥30** years who have had a hysterectomy and recently have been screened declined only 15 percentage points, and approximately 59% of these women still reported recent (in the past 3 years) Pap testing in 2010.”

